# Isolation distance between municipal solid waste landfills and drinking water wells for bacteria attenuation and safe drinking

**DOI:** 10.1038/s41598-019-54506-2

**Published:** 2019-11-29

**Authors:** Rui Xiang, Ya Xu, Yu-Qiang Liu, Guo-Yuan Lei, Jing-Cai Liu, Qi-Fei Huang

**Affiliations:** 1 ChinaState Key Laboratory of Environmental Criteria and Risk Assessment, Chinese Research Academy of Environmental Sciences, Beijing, 100012 China; 2 Research Institute of Solid Waste Management, Chinese Research Academy of Environment Sciences, Beijing, 100012 China; 3 Wuhan University of Science and Technology, Wuhan, 430081 China

**Keywords:** Environmental impact, Risk factors

## Abstract

Groundwater pollution and human health risks caused by leachate leakage have become a worldwide environmental problem, and the harm and influence of bacteria in leachate have received increased attention. Setting the isolation distance between landfill sites and groundwater isolation targets is particularly important. Firstly, the intensity model of pollutant leakage source and solute transport model were established for the isolation of pathogenic Escherichia coli. Then, the migration, removal and reduction of bacteria in the aerated zone and ground were simulated. Finally, the isolation distance was calculated based on the acceptable water quality limits, and the influence of hydrogeological arameters was analyzed based on the parameter uncertainty. The results of this study suggest that the isolation distances vary widely ranging from 106 m–5.46 km in sand aquifers, 292 m–13.5 km in gravel aquifers and 2.4–58.7 km in coarse gravel aquifers. The gradient change of groundwater from 0.001 to 0.05 resulted in the isolation distance at the highest gradient position being 2–30 times greater than that at the lowest gradient position. There was a difference in the influence of the thickness of the vadose zone. For example, under the same conditions, with the increase of the thickness of the aeration zone, the isolation distance will be reduced by 1.5–5 times, or under the same thickness of the aeration zone, the isolation distance will be significantly shortened. Accordingly, this needs to be determined based on specific safety isolation requirements. In conclusion, this research has important guiding significance for the environmental safety assessment technology of municipal solid waste landfill.

## Introduction

Groundwater pollution has become a global environmental problem that poses a continuous and serious threat to the ecological environment and human health^1–4^. Landfill leachate, which contains many toxic and harmful substances such as heavy metals, persistent organic pollutants and bacteria, has become one of the main anthropogenic sources of groundwater pollution^5^. Groundwater polluted by leachate will not only cause ecological problems such as water blooms and soil salinization, but also cause various aquagenic diseases once exposed to the human body through drinking or bathing. For example, drinking groundwater polluted by heavy metals (such as manganese and arsenic) in leachate for a long time will increase the risk of cancer and infant death, as well as induce motor and cognitive dysfunction in children^6–8^. Moreover, nitrate (NO^3−^) is ubiquitous in municipal solid waste landfills (MSWLs), and studies have shown that it is related to blue baby disorder, spontaneous abortion and increased risk of non-Hodgkin’s lymphoma^9,10^. Moreover, recent studies have revealed emerging pollutants with genotoxicity, reproductive toxicity and embryotoxicity, including hormones, antibiotics, personal care products and nanoparticles, in landfill leachate and groundwater aquifers near landfill sites^11–14^.

Although modern sanitary landfills are designed and constructed to prevent the leakage of leachate and its harmful components, accidental leachate leakage can still occur owing to damage to the geomembrane during the construction and operation of the landfill site. For example, statistics from the United States Environmental Protection Agency (USEPA) show that most landfills leak^15^. Further research has shown that 0.1%−0.4% of groundwater is polluted by landfills and industrial reservoirs. Landfill leakage may be even worse in developing countries because of the poor level of quality control and quality assurance (QA and QC) during landfill construction. For example, the hole density in geomembrane is as high as 28/hectare, which is much higher than in the United States^16^. Additionally, the degree of damage is even more serious in China. This makes it especially important to set up isolation distances between landfills and drinking water sources, especially between the scattered drinking water sources in remote areas. An appropriate isolation distance can ensure that the concentration of toxic and harmful substances after leakage will continue to decay under the interception effect of the vadose zone and the purification and dilution effect of the aquifer so that the water quality of the supply wells around the landfill site can meet the standard of safe water use^3^.

Based on the importance of the isolation distance to ensure water safety, a wide number of countries have put forward the requirements for the isolation distance from a management point of view. For example, the United States Environmental Protection Agency requires that the distance between oil storage tanks, pesticides and chemical fertilizer storage tanks and water bodies must be kept at 30.5 m^17^. Additionally, the distance between waste composting facilities and water bodies must be maintained at 61 m. Relevant standards of municipal solid waste landfills (MSWLs) require MSWL and hazardous waste landfills to maintain an isolation distance of 500 and 800 m from the surrounding residents and water bodies. In summary, it is necessary to set the isolation distance.

In this study, isolation distance is defined as the distance between the landfill and the closest drinking water well in the down-gradient direction of regional flow. Determination of the isolation distance in the above-mentioned documents takes the attenuation of heavy metals or other pollutants as the isolation target, while it seldom takes bacteria as the isolation target to calculate the isolation distance. However, previous studies have shown that leachate contains pathogenic microorganisms^18,19^. In addition, studies have shown that Escherichia coli (*E. coli*)carries pathogenic genes^20^, and the concentration of *E. coli* in landfill leachate is high (0.66 × 10^4^ MPN/100 mL)^21^. Pathogenic bacteria can contaminate water bodies; therefore, their presence must be controlled to avoid potential health hazards^19^. Accordingly, many studies have reported the extent of groundwater contamination of *E. coli* from leachate and untreated wastewater. There is also known to be a strong correlation between coliform in drinking water and diarrhea (r = 0.98)^22^. Moreover, many outbreaks of aquagenic diseases are caused by groundwater contaminated with bacteria. Significant correlations with giardiasis, the hepatitis virus, and parasitic diseases have been investigated^23^, and some studies have found that bacteria can migrate 14 kilometers in karst aquifers and 15 km in chalk aquifers^24^, while phages migrated 920 m in polluted coarse gravel aquifers and bacteria migrated 600 m in polluted gravel aquifers^25^. Although these results show that groundwater pollution caused by bacteria in leachate is widespread, there have been relatively few studies of its influence. Accordingly, it is necessary to investigate for properly determining groundwater isolation distance for landfill sites with consideration of bacteria.

This study was conducted to determine the isolation distance between landfill sites and groundwater supply wells needed to ensure that the number of pathogenic bacteria in underground wells around a landfill site are lower than the safe water standard^26,27^ under the condition of leakage.

## Materials and Methods

### Acceptable water quality standards

Many countries have put forward requirements for microbial indicators in drinking water. *E. coli* is the main indicator organism to judge whether the water quality meets the standards. For example, the EU Drinking Water Directive requires drinking water to contain <1 MPN *E. coli* and Enterococcus spp. in any 100 ml sample. New Zealand drinking water standards require drinking water to contain <1 MPN *E. coli* in any 100 mL sample43. However, the above criteria do not distinguish whether *E. coli* is pathogenic or not, and the proposed indicators are relatively broad. On the other hand, the composition of leachate produced in landfill site is complex. In order to avoid pathogenic *E. coli* contamination of groundwater by leachate leakage and the consequently problems associated with drinking water safety, the safety requirements of groundwater around landfill site should be stricter.

In addition to the standards issued by various countries, there is also a method to determine the limits of water quality indicators based on acceptable risk of infection. This method is more rigorous, so it is recommended by the World Health Organization. Based on the acceptable risk of infection (<10^–4^/person/year), the Environmental Protection Agency of the United States has established corresponding standards for different surface water treatment systems. Similarly, according to the recommendations of the World Health Organization the Netherlands has also enacted drinking water regulations^28^. The acceptable criteria for pathogenic *E. coli* in groundwater will also be determined in this paper by the risk-based method, as shown in Eq. (1):

1 $${{\rm{P}}}_{inf}\approx 1-[1+dose\frac{{({2}^{\frac{1}{\alpha }}-1)}^{-\alpha }}{{N}_{50}}]$$

Refer to Table 1 for notations and Table 2 for input variables. The solution (1) showed that the concentration of pathogenic *E. coli* in groundwater should meet <7.8 MPN/L when P_inf_ <10-4/(person/year). Therefore, 8 MPN/L was used as the minimum infection dose in this paper. (N50: Pathogenic dose causing infection in half exposed population; α: Characteristic parameters of receptor-pathogen interaction (dose-response))

**Table 1:** Notations.

Symbol	Parameter definition	Unit
P_inf_	Probability per case of *E. coli* infection based on beta-Poisson relationship	—
P	Probability of infectious *E. coli* particles	—
C	*E. coli* concentration in drinking water well	MPN/L
C_0_	Concentration of *E. coli* in the leachate	MPN/L
α	Infection risk target	<10^−4^ infections/person/year
W	Daily volume of water consumption per person	L/d/person
N	Number of ingested or inhaled microorganisms	Particle
Q	Leakage rate	m^3^/s
β_c_	Coefficient	—
h_w_	Leachate depth on the high-density polyethylene geomembranes (HDPE)	m
L_s_	Thickness of compacted clay liner	m
a	Area of defects in the HDPE	m^2^
k_s_	Hydraulic conductivity of compacted clay liner	m/s
S	Bottom area of the landfill	Ha
M	Hole density in the HDPE	holes/ha
q	Volumetric flux density of water	m/d
K	Hydraulic conductivity	—
θ	Volumetric water content	—
ψ	Water pressure potential	m
z	Depth below ground surface	m
v	Pore-water velocity	m/d
N	Van Genuchten model parameter	1 /m
θ_r_	Residual water content	—
θ_s_	Saturated water content	—
θ_e_	Effective porosity	—
α_l_	Longitudinal dispersivity	m
λ_s_	First-order virus removal rate	ln/m
μ	Virus inactivation rate	ln/d
x	Distance in direction of groundwater flow	m

**Table 2:** Parameter references.

Parameter	Subsurface media	Minimum	Maximum	Reference
Vadose zone thickness	Sand, Gravel, coarse gravel	1.0 × 10°	1.0 × 10^1^	^27^
Aquifer thickness	—	3.0 × 10°	1.0 × 10^1^	
Groundwater gradient	—	1.0 × 10^−3^	5.0 × 10^-2^	
β_c_	—	2.1 × 10^−1^	1.15 × 10°	
Parameter	Subsurface media	Mean	Standard deviation	Reference
μ	Sand, gravel, coarse gravel	3 × 10^−1^	—	^24^
α_l_	vadose zones	5.0 × 10^2^		
	saturated zones	9.8 × 10^1^	8.9 × 10^−1^	^43^
K	Sand	7.1 × 10°	3.7 × 10°	^44^
	Gravel	3.0 × 10^1^	1.7 × 10^1^	^45^
	Coarse gravel	1.5 × 10^3^	1.3 × 10^3^	^46^
θ_s_	Sand	4.0 × 10^1^	6.0 × 10^−2^	^44^
	Gravel and coarse gravel	3.0 × 10^1^		
θ_r_	Sand	4.5 × 10^2^	1.0 × 10^−2^	^44^
	Gravel and coarse gravel	2.0 × 10^2^	0	
N	Sand	2.7 × 10^0^	3.0 × 10^−1^	^44^
	Gravel and coarse gravel	2.0 × 10^0^	0	
p		5.2 × 10^1^	1.9 × 10^−1^	^47^
w		5.0 × 10^1^	2.0 × 10^−1^	^48^
r		0.4172	—	
M	Pinholes (0.1–5 mm) Holes (5–100 mm) Tears (100–10,000 mm)	4 23 10		
K_s_		1 × 10^−7^		
L_s_		0.6		
S		3		
α		0.155		
N_50_		2.11 × 10^6^		

### Concentration of pathogenic *E. coli* in leachate

Pathogenic *E. coli* was identified as the cause of various human gastrointestinal diseases, due to the existence of specific colonization factors, virulence factors and pathogenicity-related genes. At present, six pathological types of these strains have been recognized: Verocytotoxigenic *E. coli*, Enterotoxigenic *E. coli*, Enteroinvasive *E. coli*, Enteropathogenic *E. coli*, Enteroaggregative *E. coli* and Diffusely Adherent *E. coli*^20^.

Due to the complexity of pathogenic *E. coli* quantitative determination method, previous studies rarely directly determine its concentration in leachate. Only O’Toole, *et al*.^29^ reported the detection of toxic gene markers in *E. coli* isolates, but didn’t give the concentration of pathogenic *E. coli*. Considering the availability of data, the data of *E. coli* concentration in leachate were collected. According to the concentration of *E. coli*, a pathogenic ratio was introduced to estimate the concentration of pathogenic *E. coli*^20^,

2 $${C}_{PEC}={C}_{EC}\times {R}_{path}$$

where CPEC is the estimated concentration of pathogenic *E. coli* in leachate (MPN/100 ml), CEC is the measured concentration of *E. coli* in leachate (MPN/100 ml), and Rpath is the pathogenic ratio from *E. coli* to pathogenic *E. coli* (unitless). The pathogenic ratio was calculated as the proportion *E. coli* that are positive for target toxin genes in all *E. coli* isolates tested according to O’Toole, Sinclair, Malawaraarachchi, Hamilton, Barker and Leder^29^ result. Considering the uncertainty of estimates, the worst case scenario is R_path_ = 1.

### Bacteria concentration in MSWL leachate

To assess the isolation distance, we still need to know the concentration of bacteria in leachate. The operating time of MSWL, the amount of waste and age of landfill as well as the structure of landfills differ. Landfills fall into two main structures, aerobic and anaerobic. The former is an open-air landfill during its operation. The garbage has sufficient oxygen to accelerate the aerobic decomposition, stabilize the nature of the garbage quickly, settle the reactor rapidly, and produce a higher temperature (about 60 °C) during the reaction process, so that *E. coli* and other bacteria in the garbage can be eliminated. The latter is closed from the early stage of operation, and the bacteria have better living conditions. There are a large number of *E. coli* in the leachate. The concentration of *E. coli* in leachate varies greatly. As shown in Table 3, many studies have investigated coliforms and *E. coli* in leachate. Aziz, *et al*.^30^ conducted an investigation of the concentration of bacteria in MSWL leachate and found a lower limit value of 2 × 10^3^ MPN/L and an upper limit as high as 2.4 × 10^6^ MPN/L. In this study, the upper limit of the concentration was used as the source intensity parameter for the isolation distance calculation.

**Table 3:** Concentrations of *E. coli* in landfill.

Average value (MPN/100 mL)	Reference
0.15 × 10^4^	^21^
1.9 × 10^5^	^19^
24 × 10^4^	^22^
200	^22^

### The seepage rate of leachate

Although modern landfills are designed and constructed to prevent the emission of leachate, groundwater pollution caused by leachate leakage from even standard landfills still occurs frequently. Studies have shown that about three-quarters of the 55,000 landfills in the United States pollute the surrounding water^16^. And during the investigation of landfills in China, most of them were found to be leaking. This commonly occurs because some defects are inevitably introduced into high-density polyethylene geomembranes (HDPE GM) during installation and construction, which consequently forms the preferential and primary pathway of leachate leakage. Many empirical models have been developed to predict leachate leakage for different liner structures. Hydrologic Evaluation of Landfill Performance (HELP)is a quasi-two-dimensional hydrological model developed by the United States Geological Survey that is used to analyze the water balance of landfills, overburden systems, and other facilities containing soil waste. Using this model, the surface runoff, evapotranspiration, drainage, leachate collection and liner leakage of landfills can be rapidly evaluated and calculated.

In China, the engineered barrier in MSWL is typically composed of two layers of HDPE GM and compacted clay liner (CCL). For this type of barrier system, the leakage rate can be estimated according to the HELP model^31^ coupled with the empirical model developed by Giroud and Bonaparte^32^:

3 $${\rm{Q}}={\beta }_{c}[1+0.1{(\frac{{h}_{w}}{{L}_{s}})}^{0.95}]{a}^{0.1}{h}_{w}^{0.9}{k}_{s}^{0.74}\times S\times M$$

Refer to Table 1 for notations and Table 2 for input variables.

### Simulation of bacteria migration and diffusion in vadose zone and groundwater

A one-dimensional (1-D) water flow model was used to simulate vertical unsaturated flow and horizontal saturated groundwater flow. According to Nielsen, *et al*.^33^, the groundwater velocity in the 1-D unsaturated zone is simulated by:

4 $${\rm{q}}={\rm{K}}({\rm{\theta }})\cdot \frac{\partial \Psi }{\partial {\rm{Z}}}.$$

If the flow is stable, and the head pressure change $\frac{\partial \Psi }{\partial {\rm{Z}}}$ below the surface is equal to 1, the pore water velocity in the vadose zone can be calculated as:

5 $${\rm{v}}=\frac{q}{\theta }.$$

According to the van Genuchten model^34^, K(θ) is a function of moisture content θ and can be calculated using parameters N, θ_r_, and θ_s_ (Table 1). During the long-term simulation, it can be assumed that the flow rate q in the vadose zone is equal to the replenishment flow rate q in the vadose zone, after which Eqs. (4) and (5) can be solved.

The Darcy equation was used to simulate the horizontal flow, which is a function of K, groundwater gradient and effective porosity θ_e_. Effective porosity θ_e_ is calculated by subtracting θ_s_ and θ_r_^35^.

The 1-D advection-dispersion equation was used to calculate the bacteria migration in unsaturated and saturated regions, and was coupled with the first-order die-off rate for the free microbes^27^:

6 $${\log }_{10}\frac{C}{{C}_{0}}=\frac{x}{2.3}(1-\sqrt{1+4{\alpha }_{l}(\frac{\mu }{v})})/2{\alpha }_{l}$$

Refer to Table 1 for notations and Table 2 for input variables. This equation is applicable to stable groundwater flow and bacteria migration conditions. At a certain distance in the direction of groundwater flow, the initial concentration of *E. coli* (i.e., C in Eq. (6)) is calculated by C_0_, and the initial *E. coli* concentration in leachate is determined according to the practical measured data. Equation (6) was used to simulate and calculate the *E. coli* concentration after bacteria vertical leakage and migration to the bottom of the vadose zone. The concentration is then divided by the thickness of the aquifer multiplied by the width of the aquifer as the initial concentration value of bacteria migration in the saturated aquifer, after which Eq. (6) is used to simulate and predict the *E. coli* level of migration and concentration distribution in the saturated aquifer.

The bacterial index μ in different media (sand, gravel, coarse, gravel), different aquifers and vadose zones were determined according to Pang, Close, Goltz, Sinton, Davies, Hall and Stanton^24^ (Table 2). The isolation distance is simulated by using the function of the thickness of the vadose zone, groundwater gradient and hydraulic conductivity. MATLAB and statistical toolbox 2015b (The MathWorks, Inc., Natick, Massachusetts, United States) were used to compile and solve water flow and bacterial propagation.

### Calculation of isolation distance

According to the different hydrogeological parameters, the isolation distance required to realize safe drinking water under MSWL leakage conditions was simulated using the above-mentioned models. Considering the hydraulic conductivity of the aquifer under different media (sand, gravel, coarse gravel), the thickness of the aquifer was set as 3 m, the thickness of the vadose zone was 1, 3, 5, 10 and 20 m, and the groundwater gradient was 0.001, 0.005, 0.01, and 0.05. Combining different hydrogeological parameters, 144 simulation cases were obtained. The Monte Carlo method was used to simulate the influence of model parameter uncertainty on isolation distance calculation. Simulations were repeated for each case according to the Monte Carlo framework. When further computations showed no significantly different results, the isolation distance is then determined based on the concentration values at different distances along the groundwater flow direction. In addition, according to the simulation data, in order to discuss the influence of various factors on the isolation distance, the vadose zone thickness is 1 m and 10 m, and the groundwater gradient is 0.01 and 0.001, respectively.

In summary, attenuation of bacteria in the vadose zone and groundwater is affected by vadose zone and aquifer thickness, hydraulic conductivity, groundwater gradient, and so on, therefore this study focused on the influence of these parameters on the isolation distance. The basic idea is as follows: first, acceptable standards for bacteria in groundwater are determined based on the dose-response model. The model of source intensity is then selected to calculate the landfill leachate leakage. Next, the vadose zone-groundwater flow is screened and the solute transport model is used to simulate the migration, removal and reduction of bacteria in subsurface water under leachate leakage conditions and to obtain the relevant parameters of bacteria migration movement through literature research. Finally, based on the seepage source intensity-migration diffusion model established above and the risk-based acceptable bacterial concentration limit, the isolation distance of a typical landfill site was calculated, and the influence of hydrogeological parameters on this distance was analyzed.

## Results and Discussion

### Simulated isolation distance

**Figure 1:**
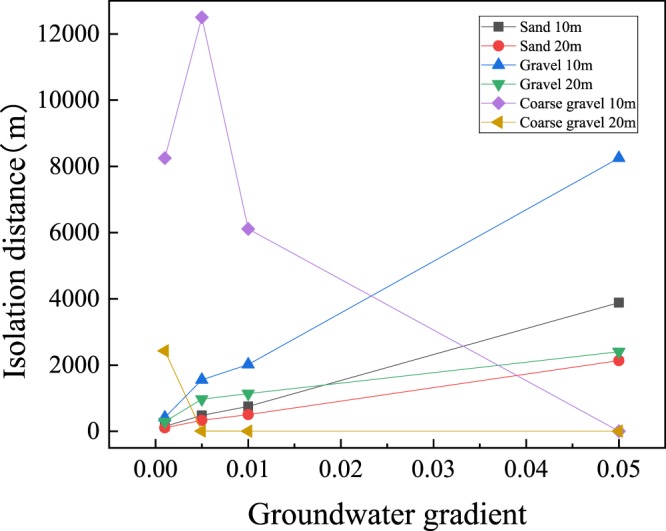
Effect of groundwater gradient on isolation distance Simulated 95th percentile setback distances from a municipal solid waste landfill required for a 12 log10 viral reduction. See Tables 2 and 3 for the input parameters; the aquifer thickness was set to 3 m.

Figure 1 and Table 4 show the isolation distance for safe use through natural attenuation of bacteria in sand, gravel and coarse gravel aquifers with a thickness of 1–20 m in the vadose zone and a groundwater gradient of 0.001–0.05. The results showed that when the groundwater gradient increased from 0.001 to 0.05, the required isolation distance in sand, gravel and coarse gravel aquifers increased from 106 m to 5.46 km, 292 m to 13.5 km and 2.4 km to 58.7 km, respectively. The isolation distance varied greatly, and the isolation distances required for different types of aquifers were quite different, with the longest distance exceeding 50 km. Leakage is a common problem in landfill sites, once it occurs, it can only rely on dilution and degradation in the vadose zone and aquifer to naturally attenuate the bacteria in leachate to meet the safety requirements^16^.

**Table 4:** Simulated isolation distance.

Vadose zone thickness (m)	Groundwater gradient	K in saturated zones (m/d)		
		Sand (5)	Gravel (30)	Coarse gravel (1300)
1	0.001	185	503	13,497
	0.005	611	2080	35,048
	0.01	979	2806	58,717
	0.05	5465	13,510	21,867
10	0.001	148	417	8248
	0.005	480	1554	12,503
	0.01	754	2016	6111
	0.05	3887	8250	<10
20	0.001	106	292	2403
	0.005	334	969	<10
	0.01	503	1140	<10
	0.05	2134	2405	<10

As shown in Table 4, when the local groundwater gradient increased from 0.001 to 0.05, the simulation isolation distance increased by 21–30 times in the sand aquifer, 1027 times in the coarse gravel aquifer and two to three times in the gravel aquifer. When the thickness of the vadose zone was reduced from 20 m to 1 m, the simulated isolation distance increased by 1.8, 2.0–5.5 and 5.0 times in different aquifers (sand, gravel and coarse gravel, respectively). The simulation results showed that when the thickness of the vadose zone was set to 10 m, the maximum isolation distance in the coarse gravel aquifer required 12 km or more. The simulation results of other circumstances showed that the isolation distance required by bacteria may be further under different aquifer conditions. For example, in polluted coarse gravel aquifers, the isolation distance can reach 58.7 km (in Table 4). Fast-flowing aquifers, such as those composed of coarse gravel, broken rocks and karst limestone, are susceptible to microbial contamination, therefore the isolation distance required is even longer^27^. However, when the gradient in the coarse gravel aquifer increased, the dilution and diffusion effect increased, causing the bacterial concentration to drop sharply and the isolation distance to be reduced^14^. For these types of aquifer media, it is proposed that the corresponding treatment plan should be made at the beginning of landfill site construction to avoid pollution of surrounding groundwater by leachate leakage.

### The influence of groundwater gradient on isolation distance

**Figure 2:**
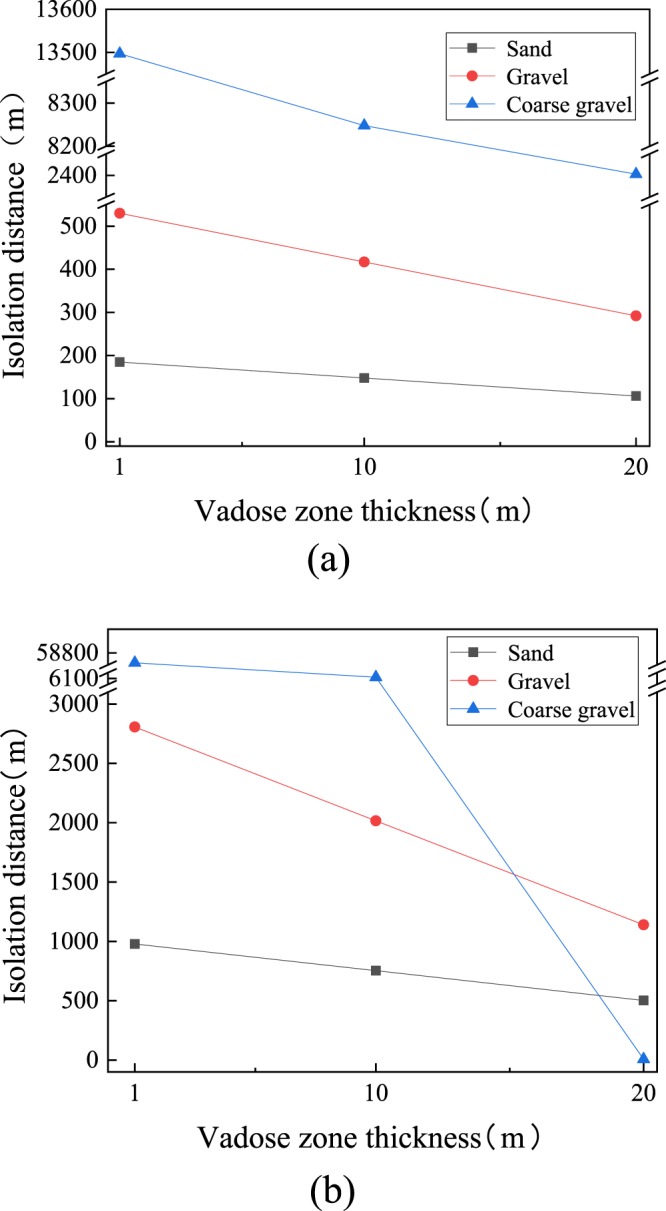
Simulated 95th percentile virus log10 reduction by passage in vadose zone and aquifer of sand, gravel, and coarse gravel as functions of setback distance for a vadose zone thickness of 1 m to 20 m; (**a**) groundwater gradient 0.01, (**b**) groundwater gradient 0.001. Aquifer thickness was set to 3 m. Input parameters are listed in Tables 2 and 3.

The required isolation distance was simulated under the condition that the thickness of the vadose zone was 1 m to 20 m respectively, and the groundwater gradient was 0.001 and 0.01, respectively (Fig. 2a,b). The simulation results showed that when the hydraulic gradient was 0.001, a smaller thickness of the vadose zone resulted in a longer isolation distance being required. When the thickness increased to 20 m, the isolation distance decreased obviously, reaching as much as 106 m. Another set of simulation result showed that when the local groundwater gradient was 0.01 and the thickness of the vadose zone exceeded 20 m, there was no need to set an isolation distance in the coarse gravel aquifer media. The increase of groundwater gradient, the diffusion and dilution effect of the bacteria in underground water was obviously enhanced, resulting in a sharp drop in concentration. In sand aquifers, the isolation distance exceeded 20 km, which is too far; therefore, the surrounding groundwater and soil will be polluted^36^.

### Influence of hydraulic conductivity on isolation distance

**Figure 3:**
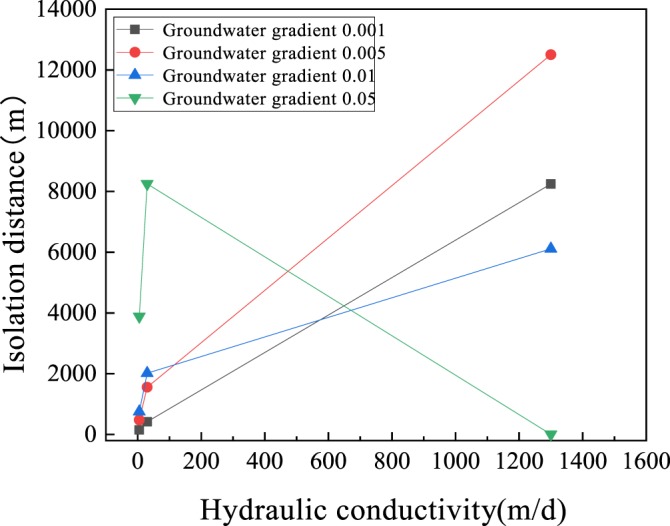
Influence of hydraulic conductivity on isolation distance (the thickness of the vadose zone is 1 m).

During the process of groundwater movement, the concentration of bacteria are not only influenced by groundwater gradient, but also by hydraulic conductivity. As shown in Fig. 3, when groundwater gradient is below 0.05, the hydraulic conductivity is directly proportional to the isolation distance. When the aquifer is coarse gravel, the required isolation distance is too far. The influence of groundwater gradient is similar to that of hydraulic conductivity, both of which are positively correlated. But, when the gradient was 0.05, the distance of groundwater flow increased greatly in the same period, while the bacterial attenuation within the same distance decreased and the isolation distance increased obviously. However, in the coarse gravel aquifer, the effect of dilution and diffusion on the bacterial concentration was much greater than the natural attenuation process, resulting in a significant reduction in the isolation distance. Considering the unique situation of landfill leakage, once leakage occurs, leachate will directly contact the vadose zone and will migrate to the underground aquifer because of gravity, causing serious groundwater pollution^37^. To prevent polluted groundwater from contaminating the surrounding geological environment, serious leakage must be avoided.

### Influence of the thickness of the vadose zone on isolation distance

**Figure 4:**
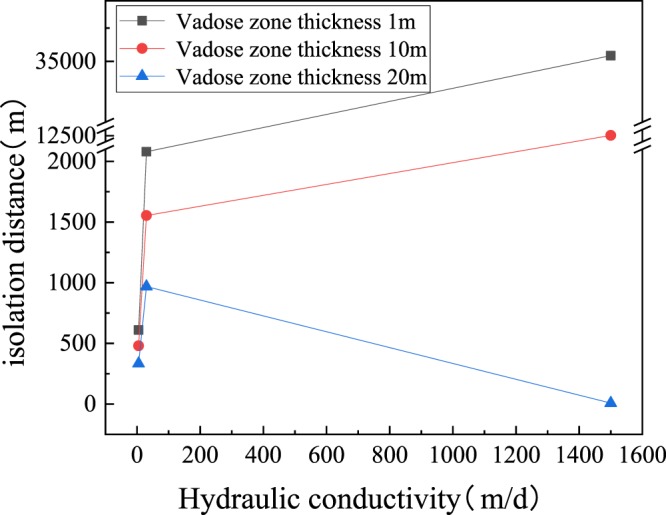
Influence of envelope thickness on isolation distance.

Groundwater gradient, hydraulic conductivity and aquifer thickness are all unchangeable hydrogeological parameters. However, the characteristics of the vadose zone can be changed by backfilling to increase the thickness, compacting to reduce the permeability coefficient, thereby enhancing its ability to degrade bacteria and lowering the requirements for isolation distance. Therefore, the influence of the thickness of the vadose zone on the isolation distance was simulated (Fig. 4). When the vadose zone thickness was 1 m, the isolation distance exceeded 35 km, which is beyond the normal planning range. When the thickness of the vadose zone increased to 20 m, the isolation distance was drastically reduced to less than 10 m. When the hydraulic conductivity exceeded 1500, the isolation distance between the gravel and the gravel aquifer increased sharply, but could be reduced to less than 10 meters in the coarse gravel aquifer. Similarly, the effect of dilution and diffusion on the bacterial concentration is much greater than the natural attenuation process in the coarse gravel aquifer, resulting in a very small isolation distance. The above-mentioned analyses show that the required isolation distance of the landfill site is very large because of limitations of the regional hydrogeological conditions, such as aquifer thickness and types and zone thickness. Nevertheless, when such a large isolation distance cannot be guaranteed because of economic, social or other factors, the requirements for isolation distance can be reduced by reforming the vadose zone to increase the thickness or reduce the permeability coefficient by compaction^38,39^.

### Model assumptions and application scope

This study focused on the influence of bacteria emissions on groundwater under MSWL leakage conditions. The seepage rate was relatively small (<10%) compared with the flow rate of the groundwater aquifer in this case, therefore it is assumed that variations in groundwater gradient because of leachate leakage can be neglected. Additionally, the study assumed that the vadose zone and aquifer medium are homogeneous and isotropic media, the seepage rate of leachate and concentration do not change with time, and the leachate and aquifer are evenly mixed throughout the aquifer thickness. However, in the actual site, the underground medium is usually non-uniform. The simulation of the isolation distance assumes that the saturated thickness of the aquifer is 1–10 m. The greater thickness is not considered because leachate usually migrates in the upper part of the saturated aquifer after leaking into the aquifer, and vertical mixing over the entire saturated thickness of the aquifer occurs only when the leakage is extremely large.

As the seepage of leachate is likely to be a continuous process, it is reasonable to assume that the aquifer thickness is subject to complete vertical mixing between 1 and 10 m. That is, when bacteria migrate from the vadose zone to the aquifer, the bacterial concentration is fully considered for dilution and diffusion effects, but the specific processes involved are not explored. To weigh the uncertainties caused by these simplified assumptions, the random properties of model variables are considered by using the Monte Carlo framework. In addition, the discharge rate and bacteria concentration in the effluent usually vary greatly with time. The uncertainty of bacteria source concentration and the limited data set, therefore the concentration in this paper refers to the data in Table 3, and the maximum value is selected for simulation.

Moreover, the migration of viruses in soil is also affected by factors such as filtration, adsorption, decay and so on. The decay of bacterial depends on many factors, such as chemical and physical conditions and microbial heterotrophic activity. For example, with the increase of temperature, the inactivation rate of MS2 bacteriophage is higher than that in moderate climatic conditions, and the temperature of groundwater is about 10°C, which is expected to have less influence^40,41^. Soil adsorption and filtration have a great impact on bacterial concentration, which not only reduces the concentration of groundwater, but also provides bacterial attachment sites. At the same time, bacteria will multiply in large quantities in the soil, which will hinder the infiltration of leachate and further reduce the concentration of groundwater^25,42^.

However, these factors are not taken into account when calculating the distance in this paper, mainly because the required isolation distance is conservative. However, from the point of view of risk control, it is acceptable to determine the isolation distance without considering these effects. Of course, further research will carefully study the impact of these factors, while incorporating the analysis of uncertainties, in order to achieve more sophisticated isolation distance settings and risk control.

If drinking water quality is considered, aquifers dominated by gravel and coarse gravel are more susceptible to bacterial pollution. This is because the high flow rate in this aquifer will reduce the filtration and adsorption capacity. If the particle size of porous media is small, the dilution of leachate is low; thus, chemical pollution and oxygen consumption will adversely affect the quality of groundwater. For instance, in terms of loamy sand aquifers and sandy loam aquifers, the isolation distance should be determined based on these parameters

## Conclusions

In this paper, a systematic health risk model was proposed to determine the effects of groundwater gradient, conductivity coefficient and aeration band thickness on the isolation distance of drinking water around landfill sites, with pathogenic *E. coli* as the target pollutant. The simulation results show that the isolation distances vary greatly among different types of aquifer media, ranging from 106 m–5.46 km in sand aquifers, 292 m–13.5 km in gravel aquifer sand 2.4–58.7 km in coarse gravel aquifers. Due to the influence of groundwater gradient and hydraulic conductivity coefficient, the isolation distance will be different for the same aquifer medium. When the simulated groundwater gradient changes from 0.001 to 0.05, the isolation distance at the highest gradient is 2–30 times larger than the lowest gradient. There was a difference in the influence of the thickness of the vadose zone. For example, under the same conditions, with the increase of the thickness of the aeration zone, the isolation distance will be reduced by 1.5–5 times, or under the same thickness of the aeration zone, the isolation distance will be significantly shortened. Consequently, the determination of isolation distance should be based on specific safety protective requirements and hydrogeological conditions. The hydrogeological parameters estimated in this study have important guiding significance for the evaluation technology of environmental safety protection of MSWL sites. Nevertheless, there are still existing plenty of factors that need to be considered during the construction and operation of the landfill site, such as the parameters of the drainage layer and the impervious layer, which need to be further studied to ensure the reasonable value of the isolation distance.
